# Supplementary material for: Chromothripsis during telomere crisis is independent of NHEJ, and consistent with a replicative origin
Source: Genome Res. 2019 May;29(5):737–49. doi: 10.1101/gr.240705.118 (PMC6499312; doi:10.1101/gr.240705.118)
Supplement: Supplemental Material [file supp_gr.240705.118_Supplemental_file_1.zip › contigs/annotated_contigs/DB92/contig.2.DB92_length_442_mean_cov_7.29411764706.docx]

**DB92_length_442_mean_cov_7.29411764706**

```
CTGCCTTCCCACGTAGCACCTTCCCTGGGGCTCCGGGGTGATGCAGGGGATGGAGGGATGGATAGGGAATCAACCAACAAACACGACAC
 >chr1:17687148-17687367 + E=3e-120
CCTCAAGTCACCTTATGCACCATAACAGGGACACACGTGGGGGCACCACCTCAGACTGGAGGGTCCGGGAAGGCCTCTGTGAGGAGGTG

GGGCTGGCACTGCCTAGGCTGAGTGGGATTCACCTTCTGG|A|CCATGTTGGTCAGGCTGGTCTCGAACTCCTGACCTAAGGTGATCCA
                                         >chr1:17807760-17807984 + E=2e-123
CCCGGCTTGGCCTCCCAAAGTGCTAGGATTACAGGCGTGAGCCACCGTGCCTGGCCACCTTTGTAAATTTACATCTTGCTTTTTGTCAA

CTAGGGGAAGGGCAGAGAGCTTTTCTTGTATCTGCTTCTTCTTAATTGGCTTCAGCTCAACATGATCCTTATGCCAAAGTTGCATGTT
```
